# Sequencing Bait: Nuclear and Mitogenome Assembly of an Abundant Coastal Tropical and Subtropical Fish, *Atherinomorus stipes*

**DOI:** 10.1093/gbe/evac111

**Published:** 2022-07-22

**Authors:** Melissa K Drown, Amanda N DeLiberto, Nicole Flack, Meghan Doyle, Alexander G Westover, John C Proefrock, Sandra Heilshorn, Evan D’Alessandro, Douglas L Crawford, Christopher Faulk, Marjorie F Oleksiak

**Affiliations:** The Rosenstiel School, University of Miami, Florida, USA; The Rosenstiel School, University of Miami, Florida, USA; Department of Veterinary and Biomedical Sciences, University of Minnesota, Minnesota, USA; The Rosenstiel School, University of Miami, Florida, USA; The Rosenstiel School, University of Miami, Florida, USA; The Rosenstiel School, University of Miami, Florida, USA; The Rosenstiel School, University of Miami, Florida, USA; The Rosenstiel School, University of Miami, Florida, USA; The Rosenstiel School, University of Miami, Florida, USA; Department of Animal Science, University of Minnesota, Minnesota, USA; The Rosenstiel School, University of Miami, Florida, USA

**Keywords:** genome assembly, mitogenome, DNA methylation, repetitive elements, population connectivity, population genetics

## Abstract

Genetic data from nonmodel species can inform ecology and physiology, giving insight into a species’ distribution and abundance as well as their responses to changing environments, all of which are important for species conservation and management. Moreover, reduced sequencing costs and improved long-read sequencing technology allows researchers to readily generate genomic resources for nonmodel species. Here, we apply Oxford Nanopore long-read sequencing and low-coverage (∼1x) whole genome short-read sequencing technology (Illumina) to assemble a genome and examine population genetics of an abundant tropical and subtropical fish, the hardhead silverside (*Atherinomorus stipes*). These fish are found in shallow coastal waters and are frequently included in ecological models because they serve as abundant prey for commercially and ecologically important species. Despite their importance in sub-tropical and tropical ecosystems, little is known about their population connectivity and genetic diversity. Our *A. stipes* genome assembly is about 1.2 Gb with comparable repetitive element content (∼47%), number of protein duplication events, and DNA methylation patterns to other teleost fish species. Among five sampled populations spanning 43 km of South Florida and the Florida Keys, we find little population structure suggesting high population connectivity.

**Significance**

Reduced sequencing costs and third generation sequencing technology have made it feasible to readily produce genomic resources for nonmodel organisms. Here we investigate *Atherinomorus stipes*, an ecologically important subtropical and tropical coastal forage fish. We present a *de novo* genome assembly and use low coverage whole genome sequencing to assess population connectivity. We detect no population structure despite a 43 km geographic spread, which will inform future fisheries and ecological studies. The *de novo* genome assembly and low coverage whole genome sequencing analysis are presented in a well annotated supplemental file S1, Supplementary Material online for use by other researchers developing genomic resources for nonmodel organisms.

## Introduction

It is estimated that 2–40 billion gigabytes of genomic data are produced each year (Stephens et al. 2015). These data result from both decreasing sequencing costs and increasing accessibility of high-throughput genomic approaches and computational analysis tools (Cosart et al. 2011; Kumar et al. 2012; Picelli et al. 2014; Schlotterer et al. 2014; Fuentes-Pardo and Ruzzante 2017; Therkildsen and Palumbi 2017; Dudchenko et al. 2018; Puritz and Lotterhos 2018; Rice and Green 2019; Gatter et al. 2020; Pallares et al. 2020; Lou et al. 2021). When population-level indices are of interest rather than individual genotypes, low-coverage whole genome sequencing (lcWGS) can be nearly as cost effective as pool-seq approaches while retaining the ability to assess individuals. Similar to pool-seq and in contrast to reduced representation approaches (e.g., genotyping-by-sequencing [Elshire et al. 2011], RADseq [Davey and Blaxter 2010]), the lcWGS approach also surveys the entire genome. Thus, lcWGS can be advantageous over reduced representation methods for population-level queries in nonmodel species, including threatened or species of interest for conservation purposes.

One potential drawback of lcWGS is the need for a reference genome (Therkildsen and Palumbi 2017). Yet, with improved accuracy in third-generation sequencing technology, including Pacific Biosciences (PacBio) and Oxford Nanopore (ONT) sequencing, it is feasible and affordable to prepare a *de novo* genome or transcriptome for nonmodel organisms (Gatter et al. 2020; Faulk 2022). Incorporating short read data can additionally mitigate long read sequencing error rates [now approximately 13% for PacBio (Ardui et al. 2018) less than 5% for ONT (Dohm et al. 2020)] and improve final assembly quality (Michael et al. 2018; Shin et al. 2019; Warren et al. 2019; Gatter et al. 2020; Chen et al. 2021). Notably, ONT is portable and can be used to sequence novel samples in the field (e.g., Johnson et al. 2017; Leggett and Clark 2017). Several recent publications and improved coding resource accessibility provide new users with detailed analysis pipelines relying on free software for genome assembly and lcWGS data analyses, which can be readily adapted (Fuentes-Pardo and Ruzzante 2017; Therkildsen and Palumbi 2017; Lou et al. 2021).

Here, we apply ONT genome assembly and lcWGS to better understand the ecology and population genetics of an abundant tropical and subtropical fish, the hardhead silverside (*Atherinomorus stipes*). This species is often included in ecosystem models and ecological surveys (Ley et al. 1999; Vaslet et al. 2015) and may be impacted by anthropogenic habitat alterations (Hernández-Mendoza et al. 2022). They are prey for several piscivorous fishes and form large schools in shallow reefs, coastal mangroves, and seagrass meadows in west Atlantic waters from Bermuda to southern Florida and Brazil. *Atherinomorus stipes* predators such as snapper, barracuda, seabream (Hammerschlag et al. 2010) are commercially important for fisheries and tourism and are often targeted among fishing communities throughout the Caribbean (Schmitter-Soto and Herrera-Pavón 2019). Therefore, understanding *A. stipes* population distribution and connectivity can improve our ability to manage and model fishery-dependent ecosystems, which ultimately can aid in conservation management in this region.

Despite their large populations and ecological importance, very little is known about *A. stipes* population dynamics. Other *Atherinidae* species have demersal eggs that attach to nearby substrate using chorionic filaments, and it is likely that *A. stipes* also have demersal eggs, limiting embryonic dispersal (Takemura et al. 2004; Nash et al. 2017). It may be expected that little genetic structure exists among *A. stipes* populations due to their ubiquity, yet egg attachment to local substrate may limit early life stage dispersal and could drive population differentiation. Previously, a study using a single mitochondrial locus (*nd2*) found high haplotype divergence but no evidence of isolation by distance (IBD) among Belize cays and Florida Keys populations (Nash et al. 2017). However, because these conclusions are drawn from a single mitochondrial locus, they may not reflect nuclear genetic diversity patterns.

In this study, we use lcWGS to investigate genome-wide population structure of five *A. stipes* populations located throughout South Florida and the Florida Keys, USA. Additionally, we use ONT sequencing to assemble the nuclear genome and mitogenome for *A. stipes*, as this was previously not available. We examine genome composition statistics to validate the assembly and compare to fish species with published genomes. These data build on existing genomic resources for *Atherinidae* species and demonstrate the utility of lcWGS paired with third generation sequencing to examine nonmodel organism genetics.

## Results

### Genome Assembly

**Fig. 1.**
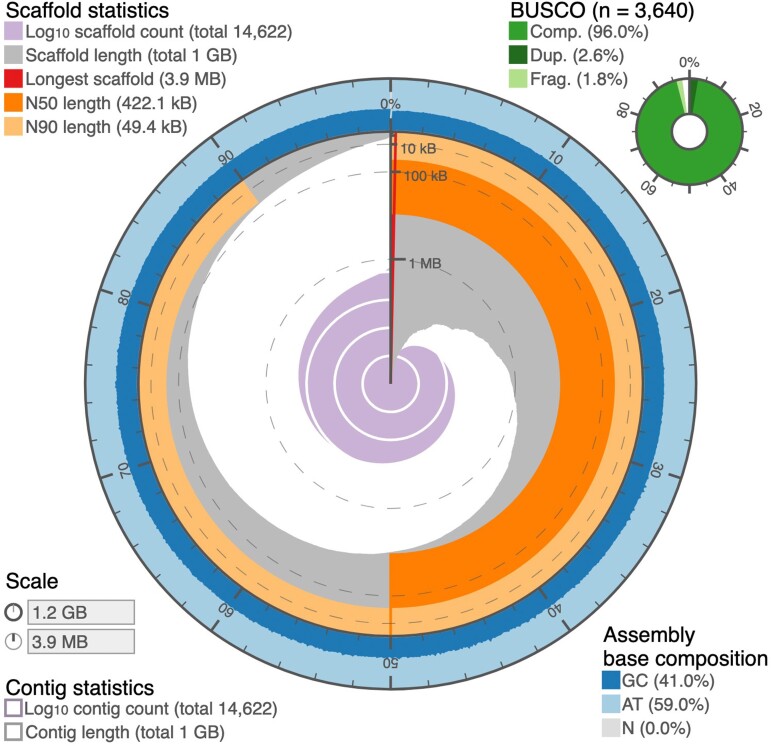
*Atherinomorus stipes* genome assembly. Snail plot summary of genome assembly statistics. From inside to outside: the central purple spiral shows log scaled scaffold count with white scale lines marking changes in order of magnitude; dark gray segments represent scaffold length distribution with plot radius scaled to the longest scaffold (red line); the orange segment represents N50 scaffold length; the light orange segment represents N90 scaffold length; outer blue and light blue rings show GC and AT percentages along the genome. Benchmarking Universal Single Copy Ortholog (BUSCO) score for the *Actinopterygii* database is in the upper right corner. To summarize and visualize genome assembly statistics, we used the software ‘assembly-stats’ (https://github.com/rjchallis/assembly-stats).

**Fig. 2.**
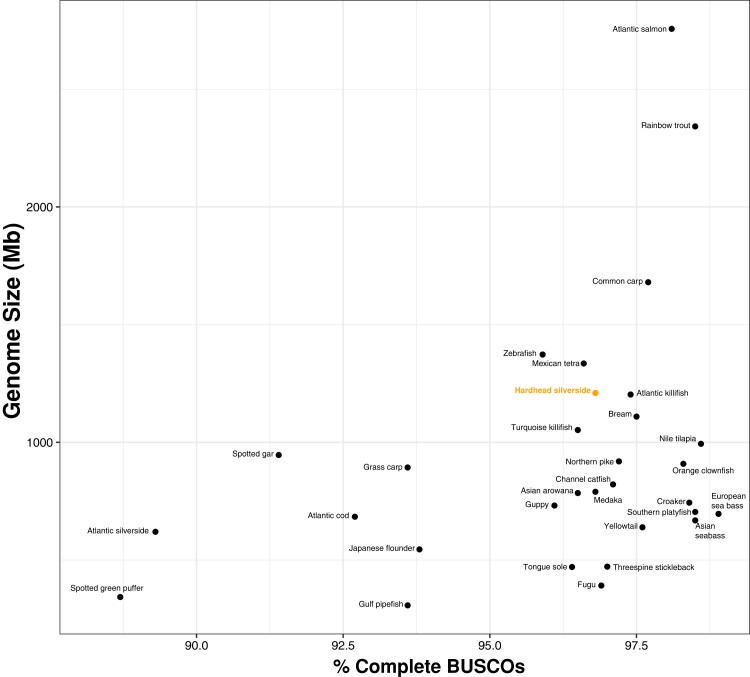
Genome size and completeness among fishes. Genome size and completeness among fishes. The *Atherinomorus stipes* genome (orange, labeled Hardhead silverside) is comparable in size and completeness to other available fish genomes (black). All additional genomes were retrieved from the National Center for Biotechnology Information (NCBI), and BUSCO scores were calculated in command line with the *Actinopterygii* marker database. All species from supplementary table S1, Supplementary Material online are labeled.

Approximately 30x genome coverage was achieved using long-read nanopore sequences for primary assembly and short read Illumina sequences for polishing. The total genome assembly length is 1,210,410,840 bp (1.2 Gb) with an N50 of 422,115 bp and 41.03% GC content (fig. 1). Although the assembly is not resolved to the chromosome level, contigs were generally large: the largest contig was 3,901,681 bp long and 82% of all contigs were less than 1 Mb in size. As a quality check, we used Benchmarking Universal Single-Copy Orthologs **(**BUSCO) scores to examine eukaryotic and *Actinopterygii* orthologous markers in the *A. stipes* genome (Simão et al. 2015). To compare *A. stipes* genome completeness to other quality assemblies, we additionally calculated BUSCO scores using the *Actinopterygii* database for 30 fishes (fig. 2; supplementary table S1, Supplementary Material online). The assembled *A. stipes* genome contains 98.0% of expected orthologs using the eukaryotic BUSCO database, and 96.8% of expected orthologs using the *Actinopterygii* database (fig. 2; supplementary table S1, Supplementary Material online). For complete *Actinopterygii* and eukaryotic markers, less than 93% are single markers (not duplicated), which suggests few misaligned or duplicated segments among contigs. Our assembly is within the expected genome size range for teleost fishes and has comparable assembly completeness to other available fish genomes (fig. 2; supplementary table S1, Supplementary Material online).

### Repeat Content and Genome Annotation

Using the *Danio rerio* database (http://www.repeatmasker.org/species/danRer.html) of known repetitive elements in RepeatMasker (Smit et al. 2013-2015), we identified 18% of the *A. stipes* genome as repetitive. This is lower than the expected repetitive DNA proportion for vertebrates (30–60%) (Lander et al. 2001; Waterston and Pachter 2002; Gibbs et al. 2004; Mikkelsen et al. 2005; Kasahara et al. 2007), and *D. rerio* is known to have many unique repetitive element families (Howe et al. 2013). Therefore, to more accurately assess repeat content, we performed *de novo* novel repetitive element detection using RepeatModeler2 (Smit et al. 2013-2015; Flynn et al. 2020). With this approach, we detected 3,164 repeat families covering 47.78% of the genome (Table 1) with the largest group of classified elements being interspersed repeats (45.22%). Of the interspersed repeats, the most common were DNA transposons (19.5%) and retroelements (7.86%).

**Table 1:** Repetitive Content

Element Classification	Number of Elements^a^	Length Occupied	Percentage of Sequence
Retroelements	295,824	95108093	7.86
SINEs	30,094	4597836	0.38
Penelope	5,670	1152119	0.1
LINEs	208,643	68631278	5.67
CRE/SLACS	0	0	0
L2/CR1/Rex	154,641	44523177	3.68
R1/LOA/Jockey	5,604	1176974	0.1
R2/R4/NeSL	3,571	1236956	0.1
RTE/Bov-B	16,904	6676757	0.55
L1/CIN4	17,007	11548094	0.95
LTR elements	57,087	21878979	1.81
BEL/Pao	4,367	3723483	0.31
Ty1/Copia	193	172536	0.01
Gypsy/DIRS1	21,734	10451783	0.86
Retroviral	6,851	3002280	0.25
DNA transposons	917,813	235992925	19.5
hobo-Activator	380,130	81335664	6.72
Tc1-IS630-Pogo	235,263	73509315	6.07
En-Spm	0	0	0
MuDR-IS905	0	0	0
PiggyBac	11,725	2044062	0.17
Tourist/Harbinger	85,041	22637991	1.87
Other (Mirage, P-element, Transib)	26,034	8063098	0.67
Rolling-circles	13,505	4833905	0.4
Unclassified	903,970	216288773	17.87
**Total interspersed repeats**		547389791	45.22
Small RNA	2,919	533198	0.04
Satellites	3,101	1397956	0.12
Simple repeats	385,531	20518992	1.7
Low complexity	62,906	4427812	0.37

Note.— Repetitive content in the *Atherinomorus stipes* genome identified using RepeatModler2 and classified using RepeatClassifier. In total, 3,164 families were identified covering 47.78% of the genome.

^a^Most repeats fragmented by insertions or deletions have been counted as one element.

To assess ascertainment bias between genic and intergenic regions and compare predicted genic regions among fishes, we used *AUGUSTUS* gene annotation software (Stanke and Waack 2003; Keller et al. 2011). Using *D. rerio* trained models, *AUGUSTUS* gene annotation software identified 46.0% of the genome as genic versus intergenic regions with 89.8% of bases in genic regions having 10x or greater coverage. Genic regions were targeted for coding region and protein identification with 51,144 nucleotide coding genes and 51,142 amino acids detected. Within predicted coding regions (all but two genic regions), 288,543 exons were identified. Our four billion sequence reads were split equally between genic and intergenic regions with 48.6% landing within genic regions (no significant enrichment, 1.05-fold intergenic:genic). Of the 51,142 putative genes, orthofinder assigned 35,895 (70%) to an orthogroup (i.e., identified as duplicate). The magnitude of *A. stipes* specific gene duplication is comparable to other fish species (supplementary fig. S1, Supplementary Material online).

### DNA Methylation

We used Megalodon (https://github.com/nanoporetech/megalodon) to detect changes in raw nanopore sequencing signal at cytosine bases where methylation occurs in 5-methylcytosine (5-mC) and 5-hydroxymethylcytosine (5-hmC) contexts. For these methylation analyses, flow cell runs were analyzed separately as technical replicates. For both runs, we examined sites with more than 10 reads and an average depth of 99.3 reads per site and found 5-mC DNA methylation of 78% and 5-mHC below 1%. Results were highly repeatable between runs despite subtle differences in sequencing statistics between runs (supplementary table S2, Supplementary Material online), as expected based on prior studies using Megalodon (Liu et al. 2021).

### Mitogenome Assembly

**Fig. 3.**
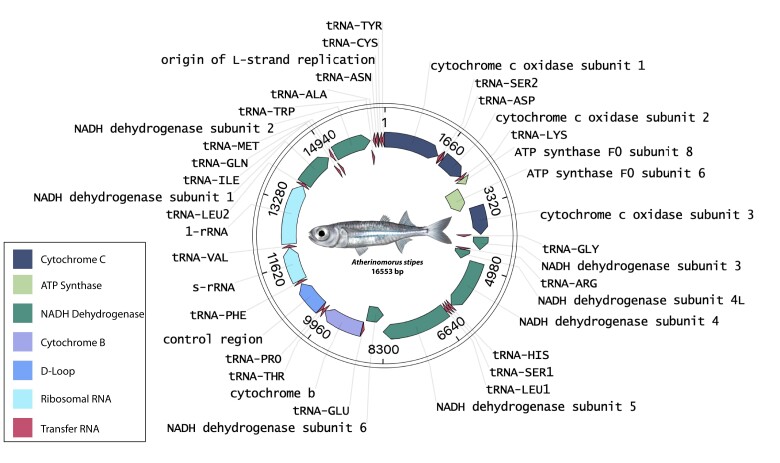
*Atherinomorus stipes* mitogenome. Total mitogenome size is 16,553 bp and contains 22 tRNAs, seven NADH subunits, four COX subunits, two ATP synthase subunits, and one D-loop control region. Mitogenome was annotated through the MITOchondrial genome annotation server (MITOS2) website with the start site manually set to COX1 and visualized with open vector editor.

We expected to have relatively high coverage of the mitochondrial DNA, which is about 16 kb in size, due to the high mitochondrial DNA to nuclear DNA ratio found in most animals. The assembled *A. stipes* mitogenome is circular with a total size of 16,553 bp, containing 22 tRNAs, seven nicotinamide adenine dinucleotide (NADH) subunits, four cytochrome oxidase (COX) subunits, two adenosine triphosphate (ATP) synthase subunits, and one D-loop control region (fig. 3). The sequence is 79.13% similar to *Menidia menidia* (11,687 bp) and 84.31% similar to *A. lacunosus* (16,552 bp), an Indo-Pacific silverside.

### Connectivity Among Populations

**Fig. 4.**
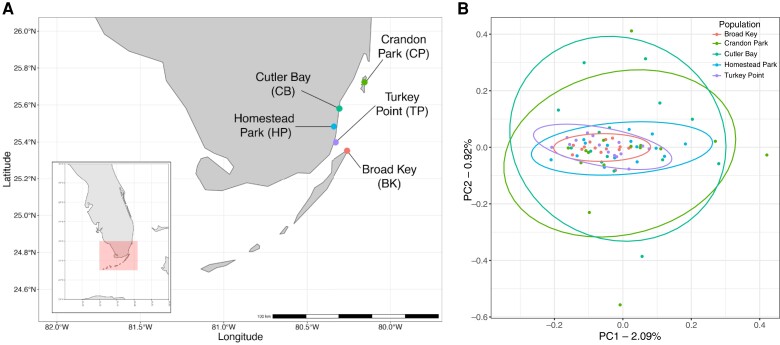
*Atherinomorus stipes* population map and principal component analysis. (*A*) Five sites were sampled in South Florida and the Florida Keys. Sites include (North to South) Crandon Park (green), Cutler Bay (teal), Homestead Park (blue), Turkey Point (purple), and Broad Key (red). Map generated in R. (*B*) Based on 266,731 nuclear SNPs among 75 individuals from five coastal *Atherinomorus stipes* sites, the sampled sites show no population structure. PCA was generated using PCAngsd and plotted in R.

Using our assembled genome, we mapped lcWGS reads from *A. stipes* individuals collected form five South Florida populations spanning 43 km (fig. 4*A*; supplementary table S3, Supplementary Material online) and calculated genotype likelihoods for variable sites using ANGSD (Korneliussen et al. 2014). After filtering (supplemental file S1, Supplementary Material online), we used 266,731 single nucleotide polymorphisms (SNPs) to assess population connectivity among 75 individuals (N = 15 per population) with principal component analysis in PCAngsd (Meisner and Albrechtsen 2018). The first two principal components (PCs) explained 2.09% (PC1), and 0.92% (PC2) of variance among individuals (fig. 4*B*). All populations clustered into a single group when using any of the first ten PCs examined, which together accounted for 10.32% of variance among individuals (supplementary fig. S2, Supplementary Material online).

## Discussion

We use ONT to rapidly assemble a high-quality genome (based on BUSCO completeness and N50) for a nonmodel organism without any existing genomic resources. Our pipeline relies on open-source software and includes repetitive element detection and classification, DNA methylation and hydroxymethylation quantification, genic region prediction and annotation, and orthologous protein region identification. Oxford nanopore technology is frequently used in metagenomics (Loit et al. 2019; Overholt et al. 2020; Kerkhof 2021) and for vertebrate genome assembly (Jansen et al. 2017; Tan et al. 2018; Dhar et al. 2019; Bian et al. 2020; Johnson et al. 2020; Xu et al. 2021). Here, we provide methods (supplemental file S1, Supplementary Material online), that use fairly low coverage (∼30x) combined ONT and Illumina sequencing for vertebrate genome assembly and genomic investigations. This is similar to assembly methods in Gatter et al. (2020) emphasizing how genomic resources can be readily produced for nonmodel organisms.

### *Atherinomorus stipes* Genome

Despite relatively low coverage (∼30x), our genome assembly is of reasonable size and quality compared to other available fish genomes (fig. 2; supplementary table S1, Supplementary Material online). The *A. stipes* assembly has a total size of approximately 1.2 Gb with 41% GC content and an N50 of 422,115 bp (fig. 1). This is about twice the genome size of an available close relative, Atlantic silverside (*M. menidia*), which is roughly 620 Mb (Tigano et al. 2021), and similar in size to Atlantic killifish (*Fundulus heteroclitus*) and zebrafish (*Danio rerio*) (fig. 2; supplementary table S1, Supplementary Material online). We also found protein duplications to be comparable in magnitude to those present among other fishes (supplementary fig. S1, Supplementary Material online), suggesting that there is not a lineage specific genome duplication contributing to genome size variation among species. Instead, we suggest that genome size variation is due to repeat content variation, which we identified to be 47.78%, ∼40% higher than the 17.73% detected in *M. menidia* and among the highest present in any fish (Yuan et al. 2018). This is reasonable as repetitive element content is known to increase with increasing genome size among fishes (Yuan et al. 2018) and other organisms (Lee and Kim 2014; Sessegolo et al. 2016; Blommaert et al. 2019). Notably, *Atheriniformes* are within the same clade as *Cyprinidontiformes* (Hughes et al. 2018), and available data on repetitive element content among cyprinodont fishes show similar genome size and repetitive element content as we have identified in *A. stipes* (Yuan et al. 2018).

For further comparison, we examined the *A. stipes* epigenome by identifying CpG sites with 5′-methylcytosine (5-mC) and 5′-hydroxymethylcytosine (5-mhC) modifications. Nanopore uniquely allows epigenetic modifications including DNA or RNA methylation in 5-mC and 5-mhC contexts to be detected from direct DNA and RNA sequencing, which is an improvement over other methods that cannot differentiate 5-mC and 5-mhC and leads to overestimation of 5-mC presence (Metzger and Schulte 2016; Wanner et al. 2021). With ∼15x genome coverage we identified an average DNA methylation among CpG sites of ∼78% 5-mC and ∼0.3% 5-mhC in two runs (supplementary table S2, Supplementary Material online). These 5-mC and 5-hmC values are similar to those found in zebrafish (78% 5-mC methylation and 2.3% 5-mhC) (de Mendoza et al. 2019) and are in the expected range for vertebrate genome 5-mC and 5-mhC values (Jabbari et al. 1997; Jiang et al. 2014; Keller et al. 2015; Colwell et al. 2018). To our knowledge, no prior studies have used Nanopore sequencing to examine DNA methylation in fish (Metzger and Schulte 2016). Nanopore sequencing may be beneficial in future studies examining DNA methylation to reduce time and cost by preventing the need for sample pre-treatment. These methods could be applied in fish to further study DNA methylation variation with habitat temperature (Varriale and Bernardi 2006), across generations or populations (Hu et al. 2021; Kelley et al. 2021), or in response to environmental stressors (Aluru et al. 2011). Due to the portability of Nanopore, investigations of epigenetic variation among otherwise genetically indistinguishable populations could enhance our understanding of species’ environmental responses.

### *Atherinomorus stipes* Population Connectivity

The five *A. stipes* populations examined here are spread across 43 km of coastal habitat where salinity, anthropogenic influence, and temperature fluctuate widely (fig. 4*A*). While higher salinity has previously been correlated with greater abundance for this species, it does not seem to influence genetic structure among these populations (fig. 4*B* [Ley et al. 1999]). Despite distance and habitat variation, we do not observe population structure among these populations (fig. 4*B*). Similar to previous observations using the mitochondrial locus *nd2,* IBD is not supported in this data set (Nash et al. 2017). Although it is likely these fish lay demersal eggs, this does not impact population connectivity. Instead, it appears gene flow is not restricted among these southeast Florida populations. This may be due to high migration and low-site fidelity, or larval distribution by currents, resulting in a single admixed population.

Insights into population structure and connectivity are important for fisheries modeling. Typically, fisheries incorporate species information based upon stock units, which are discrete populations (Ovenden et al. 2015). Incorporating genetic data in ecological and fisheries modeling can improve how a stock is defined in terms of population and community structure (Overcast et al. 2019). Additionally, genetic data can improve resolution of ecological factors, recruitment, connectivity, signatures of selection and adaptive response (Bernatchez et al. 2017). *Atherinomorus stipes* is often included in fisheries models in South Florida and the Florida Bay (Flaherty et al. 2013, Smith et al. 2021). With our lcWGS data we can conclude that among the sampled southeast Florida sites, *A. stipes* is not impacted by habitat fragmentation. The region represented in this study can be included as a single population stock, which was previously uncertain based on limited data. Overall, incorporating lcWGS allows for greater knowledge that has many applications for improving many fields, including fisheries management. Future studies encompassing more of *A. stipes’* range, throughout the Caribbean, Gulf of Mexico, and northeast coast of South America would further inform the degree of genetic connectivity among populations.

## Conclusions

Our study builds on existing genomic resources for teleost fish and provides a novel genome for *Atherinidae* species. The *A. stipes* genome assembly is similar in completeness and quality to other available fish genomes, and future studies should consider its inclusion in comparative genomics work, especially among fishes. In addition, we show that *A. stipes* individuals sampled across this 43 km range do not exhibit significant population structure. As these fish are an important prey item for several economically important species, we suggest that future ecological models could consider *A. stipes* found across this spatial scale as a single interbreeding population with little fragmentation or structure. However, any conclusions about population structure outside of the range presented here would require further testing, which will be possible with the genomic resources developed here. Overall, we highlight the importance and simplicity of increasing genomic resources and genome sequence data in nonmodel organisms. Doing so, particularly for commercial fishery species, can increase understanding of threatened habitats and allow for improved modeling resolution and conservation practices.

## Methods

### Sample Collection

Fin clips from adult *A. stipes* individuals were collected from five populations in south Florida including: Crandon Park (CP, 25° 43′23.34″N 80° 9′16.6176″W), Cutler Bay (CB, 25°34′46.776″N 80°18′18.036″W), Turkey Point (TP, 25°23′47.9″N 80°19′39.1″W), Homestead Park (HP, 25°28′59.1″N 80°20′21.6″W), and Broad Key (BK, 25°21′04.7″N 80°15′32.4″W) (fig. 4*A*; supplementary table S3, Supplementary Material online). For one individual, whole tissues (except stomach and gut) were homogenized in NaNH_4_ buffer and DNA extracted using phenol-chloroform isoamyl (PCI) alcohol precipitation, spooling the DNA sample with a glass pipette, and storing in 0.1X TE buffer. For all other individuals, fin clips were digested in chaotropic buffer with 10% proteinase K overnight and DNA was extracted using magnetic beads. Procedures were approved by the University of Miami Institutional Animal Care and Use committee. Fish collected within Biscayne National Park were permitted under permit # BISC-2021-SCI-0019.

### Sequencing

Genomic DNA was shipped on ice to the University of Minnesota, prepared with the SQK-LSK-109 (run 1) or SQK-LSK-110 (run 2) kit, and sequenced on an in-house ONT MinION instrument. Each sequencing run used 2 ug of DNA as input and took place over 2 days with one wash (wash kit 004) and reloaded with DNA from the original library prep.

For population genetics analysis and whole genome polishing, an additional 76 samples (15 per population, ONT individual at 10X) were prepared for 150 bp paired end sequencing on an Illumina HiSeq3000 (Genewiz LLC, New Jersey, USA). Briefly, a recombinant Tn5 was used as in (Picelli et al. 2014) with 15 cycles of PCR amplification, similar to the Illumina Nextera XT DNA library preparation kit. Final libraries were size-selected to 300–700 bp fragments using SPRI magnetic beads. Dual barcodes for each individual and sequencing primers used for library preparation are available in supplementary table S4, Supplementary Material online.

### Analysis Pipeline

A complete analysis pipeline and description of methods used are available in supplemental file S1, Supplementary Material online. Briefly, the *A. stipes* genome was assembled using Nanopore reads from one Crandon Park individual with Flye (v2.9) (Kolmogorov et al. 2019) and polished once with Racon (v1.4.20) (https://github.com/isovic/racon), once with Medaka (v1.4.4) (https://github.com/nanoporetech/medaka), and four times with Pilon (v1.24) (Walker et al. 2014) using mapped and trimmed (to remove Illumina adapters and low-quality bases) short read sequences from the same individual. We used the genome assembly for comparative genomic analyses including assessment of genome quality and completeness (BUSCO v5.2.2 [Simão et al. 2015]), repeat content (RepeatModeler2 [Flynn et al. 2020]), gene prediction (AUGUSTUS v3.3.3 [Stanke and Waack 2003]), and orthologous protein presence (Orthofinder v2.5.4 [Emms and Kelly 2019]). We assembled the mitogenome with GetOrganelle (Jin et al. 2020) using 119,584,855 short-read sequence reads from the same individual that was used for the nuclear genome assembly. The *M. menidia* (Atlantic silverside) mitogenome was used as a seed database (Lou et al. 2018; Jin et al. 2020). For population genetic analysis, we processed Illumina reads following a standard lcWGS pipeline as in (Lou et al. 2021) (supplemental file S1, Supplementary Material online).

## Supplementary Material

evac111_Supplementary_Data

## Data Availability

All sequence data and genome assemblies have been deposited in NCBI. The *Atherinomorus stipes* genome assembly is available at NCBI Bioproject PRJNA814500. The *A. stipes* mitogenome assembly is available at NCBI accession OM974321. Raw Nanopore and Illumina sequence data is available in the NCBI SRA under Bioproject PRJNA814490.
