# Supplementary material for: Sequencing Bait: Nuclear and Mitogenome Assembly of an Abundant Coastal Tropical and Subtropical Fish, Atherinomorus stipes
Source: Genome Biol Evol. 2022 Jul 22;14(8):evac111. doi: 10.1093/gbe/evac111 (PMC9348626; doi:10.1093/gbe/evac111)
Supplement: evac111_Supplementary_Data [file evac111_supplementary_data.zip › Hardhead-Supplemental_File_1_revised.pdf]

Hardhead

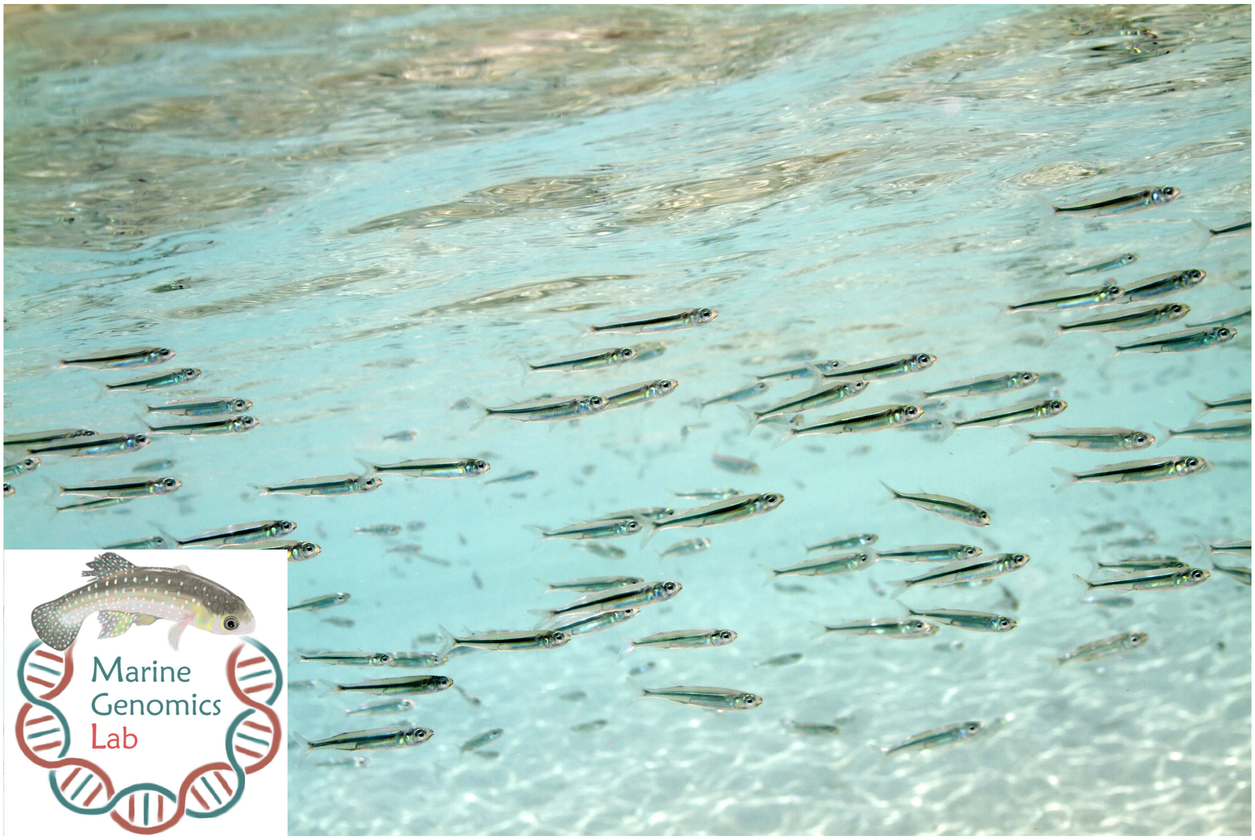

Hardhead Silverside Genomic Analyses

▼ Contents

Overview

Draft Genome Assembly

Computational environment and software

Read generation and genome assembly

Assembly quality control and statistics

Methylation analysis

Genome annotation

Repeat analysis

Protein annotation and duplication

Draft Mitochondrial Genome Assembly

LcWGS data processing

Preparation and software

Trim adapters and low quality sequences

Merge overlapping reads using FLASH

Map reads to the genome and filter

LcWGS data analyses

Overview

We assembled a draft genome for hardhead silverside (*Atherinomorus stipes*) using nanopore sequences polished with Illumina short read sequences from the same individual and used this genome to map low coverage whole genome sequencing (lcWGS) reads from hardhead silverside samples collected in Biscayne Bay, FL. Next, we used the mapped reads to call genotype likelihoods. Finally, we used these genotype likelihoods to explore hardhead silverside population structure in Biscayne Bay.

All data visualizations were done in RStudio (v1.4.1106) unless otherwise noted. All Illumina sequences are available at NCBI SRA: PRJNA814490. The pipelines for the described data analyses are detailed below.

We ran our code on a sequencing computer located in the Faulk Lab at the University of Minnesota or on the University of Miami’s high performance computing system through slurm job submission. Many commands require high performance computing and should not be attempted on a standard local machine (*e.g.,* a personal laptop). When possible, we ran our code in parallel using GNU parallel. Unless otherwise indicated, default parameters were used.

Draft Genome Assembly

Computational environment and software

A single hardhead silverside individual was used for nanopore sequencing on the Oxford Nanopore MinION instrument. Nanopore basecalling used Guppy, a data processing toolkit run from the command line that contains the Oxford Nanopore Technologies' basecalling algorithms and several bioinformatic post-processing features. Using an external GPU (graphics processing unit) to basecall with Guppy dramatically increases basecalling speed over using CPU (central processing unit). The sequencing computer located in the Faulk Lab at the University of Minnesota was built with both GPU and CPU.

This sequencing computer was built with a NVIDIA GeForce 2080Ti graphics coprocessor (GPU) with 4352 CUDA cores and an AMD Ryzen 3900x processor (CPU) with 12 threads, 24 cores, 64 Gb RAM, and a 1 Tb SSD (solid state drive). It used the Ubuntu Linux 20.04 operating system with a Cinnamon desktop environment. The GPU was tested for defects using gpu-burn. The computer was remote monitored with NoMachine (https://www.nomachine.com). We monitored the CUDA cores with nvtop (https://github.com/Syllo/nvtop).

Read generation and genome assembly

Fastq files were generated from Oxford Nanopore MinION instrument and called with Guppy (v5.0.11). We used the GPU enabled version of guppy in concert to enable live basecalling for adaptive sampling and for post-hoc basecalling with the high accuracy model (accuracy = 97.8 using r9.4.1 flow cell chemistry). All resulting fastq files were concatenated and used for assembly with Flye (v2.9) (Kolmogorov, Yuan et al. 2019) with the --nano-raw parameter specified. The assembled genome was polished once with Racon (v1.4.20) (https://github.com/isovic/racon), once with Medaka (v1.4.4) (https://github.com/nanoporetech/medaka), and four times with Pilon (v1.24) (Walker, Abeel et al. 2014) using mapped and trimmed (to remove Illumina adapters and low-quality bases as described below for lcWGS processing) short read sequences from the same individual.

▼ Overview of genome assembly and processing

- Called nanopore bases with Guppy
- Assembled draft genome with Flye
- Polished the assembly with nanopore reads
    - Racon
    - Medaka
- Polished the assembly with Illumina short reads
    - Pilon (4x)
- Assessed the genome assembly quality
    - BUSCO
    - Kraken2
    - Quast

▼ Software Used

- Guppy
- Flye
- Racon
- Medaka
- Pilon
- BUSCO
- Megalodon
- Kraken2
- Quast
- RepeatModler2
    - RepeatClassifier

- RepeatMasker

▼ CODE

```
# Basecalling with Guppy v5.0.11

guppy_basecaller --config dna_r9.4.1_450bps_hac.cfg --device "cuda:0" \
--nested_output_folder --recursive --input_path /path/to/fast5/ \
--save_path /path/to/output/location


# Assembly with Flye v2.9

flye --nano-raw passed_basecalls_ALL.fastq --out-dir Flye_assembly_ALL


# Assembly polishing - Racon v1.4.20

racon -m 8 -x -6 -g -8 -w 500 -t 14 passed_basecalls_ALL.fastq \
mapping.sam assembly.fasta > racon.fasta


# Assembly polishing - Medaka v1.4.4

medaka_consensus -i passed_basecalls_ALL.fastq -d racon.fasta -o racon_medaka \
-t 6 -m r941_min_hac_g507


# Assembly polishing - Pilon v1.24 (repeated four times)

bwa mem -t 14 consensus.fasta fwd_All.fq.gz rev_All.fq.gz | samtools view - -Sb \
| samtools sort - -@14 -o mapping.sorted.bam

samtools index mapping.sorted.bam
java -Xmx120G -jar pilon-1.24.jar --genome consensus.fasta --fix all \
--changes --frags mapping.sorted.bam --threads 14 --output pilon_round1 \
| tee round1.pilon
```

Assembly quality control and statistics

To assess genome completeness, BUSCO (v5.2.2 with hmmsearch v3.1 and metaek v5.34) (Simão, Waterhouse et al. 2015) score was calculated in genome mode against the eukaryota_odb10 BUSCO database and the Actinopterygii_odb10 BUSCO database. In addition, the genomes of 30 fish species were retrieved from NCBI and used for BUSCO analysis (Table S1). To summarize and visualize genome assembly statistics we used the software 'assembly-stats'. Results from assembly-stats were visualized by loading the result into the assembly-stats.html run on a local webserver.

▼ CODE

```
# Quality Control - BUSCO v5.2.2
# Dependencies: hmmsearch v3.1 and metaeuk v5.34

busco -m genome -l eukaryota_odb10 -i pilon_round4.fasta -o busco_euk
busco -m genome -l actinopterygii_odb10 -i pilon_round4.fasta \
-o busco_fish_database

# Quality Control - Kraken2
```

```
kraken2 --db /mnt/bc91d872-d479-41b6-b994-c521c27d41cf/kraken2/k2_pluspf16gb/ \
--threads 23 --use-names --report HHSilverside.kraken2.report.txt \
--output HHSilverside.kraken2.out.txt aaa.fastq


# Quality Control - QUAST v5.0.2

quast.py -t 14 -o quast_compare2 assembly.fasta racon.fasta consensus.fasta \
pilon_round1.fasta pilon_round2.fasta pilon_round3.fasta pilon_round4.fasta
```

Methylation analysis

DNA methylation in both 5-methylcytosine (5-mC) and 5-hydroxymethylcytosine (5-mhC) contexts was called from raw Nanopore sequence data using Megalodon (v2.3.3) (https://github.com/nanoporetech/megalodon). Results from each flow cell are reported separately as technical replicates.

▼ **CODE**

```
# Methylation Analysis with Megalodon v2.3.3

megalodon / no_sample/20211006_1711_MN34646_FAQ58375_09c6c0a6/fast5_pass/ \
--outputs basecalls mappings mod_mappings mods per_read_mods \
--reference ~/Desktop/HHSilverside-assembly/astipes_fishgenome_pilon4.fasta \
--devices 0 --processes 23 --guppy-server-path ~/ont-guppy/bin/guppy_basecall_server \
--output-directory HHSilverside10kit_meth/ --guppy-params "-d /home/cfaulk/ont-guppy/data/rerio/basecall_mod
els/" \
--guppy-config res_dna_r941_min_modbases_5mC_5hmC_v001.cfg --mod-binary-threshold 0.8 --overwrite 2


## Post process megalodon output
# The following commands were used to get *only* CG methylation sites:

megalodon_extras modified_bases create_motif_bed --motif CG 0 \
--out-filename CG-motif.bed ~/Desktop/HHSilverside-assembly/astipes_fishgenome_pilon4.fasta

## Intersect bed files to add 5mC values to them

bedtools intersect -a modified_bases.5mC.bed -b CG-motif.bed > CG-motif.5mC.methvalues.bed
bedtools intersect -a modified_bases.5hmC.bed -b CG-motif.bed > CG-motif.5hmC.methvalues.bed


# (last two columns are "depth of coverage" & "percentage of methylation")

## Use awk to average the methylation column for whole genome methylation.
# This case uses only positions where the count is > 10 reads.

awk '$10>10 {total +=$11; count++} END{print total /count}' CG-motif.5mC.methvalues.bed
```

Repeat analysis

Repetitive DNA content was identified using RepeatModeler2 (Smit, Hubley et al. 2013-2015, Flynn, Hubley et al. 2020) with the curated Dfam database release 3.5 July 2021 (Storer, Hubley et al. 2021), combined with the Repbase RepeatMasker database downloaded on October 26, 2018. The repeats were classified using RepeatClassifier against the same combined Repbase and Dfam database and masked using RepeatMasker to annotate repetitive DNA in the *A. stipes* assembly.

▼ **CODE**

```
# Repeat Content Analysis with RepeatModler2, RepeatClassifier, and RepeatMasker
## First use RepeatModler2 to predict repetitive element families and classify with
# RepeatClassifier. Then run RepeatMasker as below to mask REs.

RepeatMasker v4.1.2-pl -pa 24 -s [sensitive mode] [using rmblast 2.11.0+] \
-lib HHSilverside-families.fa astipes_fishgenome_pilon4_masked.fasta
```

Genome annotation

AUGUSTUS (v3.3.3) (Stanke and Waack 2003, Keller, Kollmar et al. 2011) was used to generate *de novo* gene annotation using zebrafish trained models with the soft masking option to ignore repetitive elements. Nucleotide and amino acids hits were extracted from the resulting gff file and identified using the National Institute of Health non-redundant database for blastp searching.

▼ CODE

```
# Call augustus with softmasking turned on to ignore repeats

augustus --species=zebrafish astipes_fishgenome_pilon4.fasta \
--softmasking=1 > augustus-predictions-softmasked.gff

# Extract the nucleotide and amino acid hits from the gff file into fasta files.
./augustus-master/scripts/getAnnoFasta.pl augustus-predictions-softmasked.gff \
--seqfile=astipes_fishgenome_pilon4.fasta

# Get the NIH nr (non-redundant protein database for blastp searching)
wget 'ftp://ftp.ncbi.nlm.nih.gov/blast/db/nr.*.tar.gz'
cat nr.*.tar.gz | tar -zxvi -f - -C .

# use Diamond to speed up blasting with blastp to blast proteins against NIH nr database to identify their type
diamond -prepdb -d nr
time diamond blastp --query augustus-predictions-softmasked.aa \
-d nr -p 22 -v -o \
augustus-predictions-softmasked.diamond.out --max-target-seqs 1 -f 6 \
qseqid sseqid pident length mismatch gapopen qstart qend sstart \
send evalue bitscore slen nident mismatch stitle salltitles
```

Protein annotation and duplication

Orthofinder (v2.5.4) (Emms and Kelly 2019) was used to calculate gene duplication events and orthogroups within *A. stipes* through comparison to 12 other fishes.

Draft Mitochondrial Genome Assembly

The mitogenome was assembled in GetOrganelle (v1.7.5.1) (Jin, Yu et al. 2020) using Illumina short read sequences (adapter and quality trimmed as for lcWGS data, described below) with the *Menidia menidia* mitogenome from the Jekyyl Island population (Lou, Fletcher et al. 2018) as a seed database. Mitogenome annotation and manual rearrangement to start the circular mitogenome at cytochrome oxidase 1 (COX1) was done as in (Wanner, Larsen et al. 2021) with MITOS2 and open vector editor. Similarity among species in mitogenome sequence was assessed using NCBI blast.

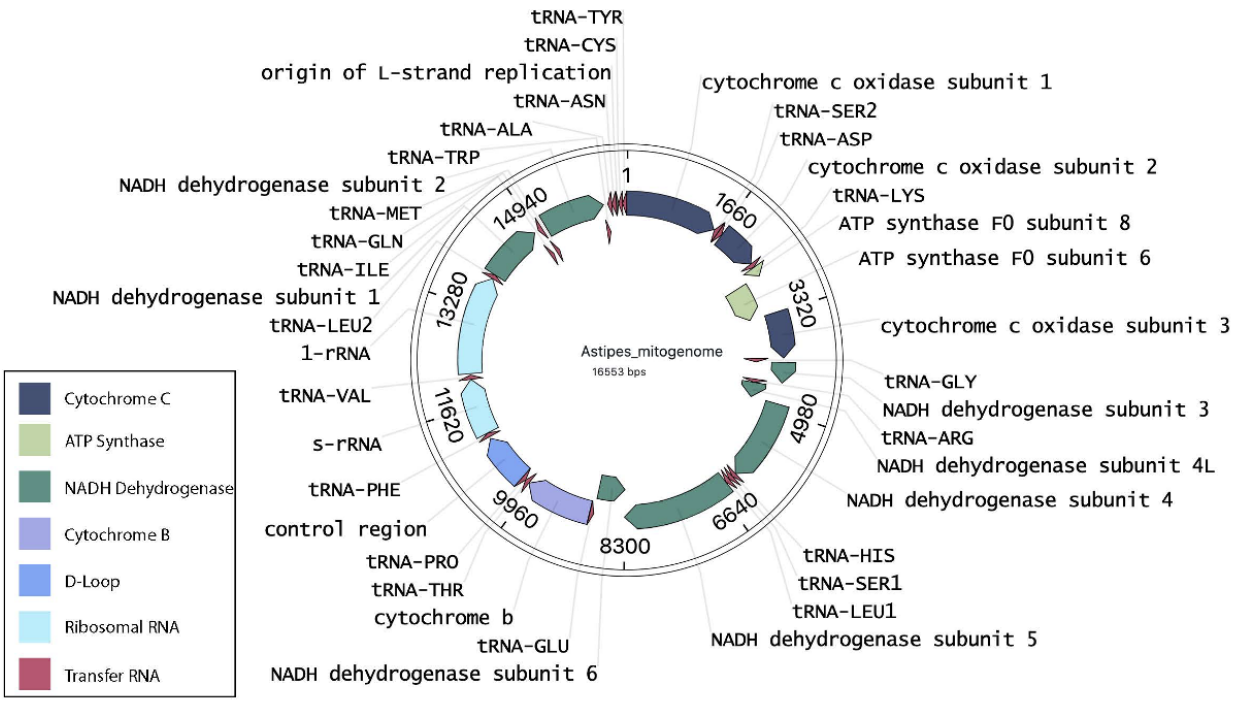

▼ **CODE**

```
# Mitogenome Assembly with GetOrganelle

get_organelle_from_reads.py -s menidia_JI_mito.fasta -1 illumina_reads_fwd.fasta \
-2 illumina_reads_revs.fasta -t 1 -F animal_mt -R 10 -k 21,45,65,85,105 \
-o output_mitogenome
```

lcWGS data processing

Preparation and software

To prepare samples for population genetic analyses, we followed a standard lcWGS pipeline as in (Therkildsen and Palumbi 2017).

▼ Overview of raw sequence processing

- Removed adapters and low-quality bases using Trimmomatic (v0.39) (Bolger, Lohse, et al. 2014).
- Merged overlapping reads using FLASH (v1.2.11) (Magoč and Salzberg 2011).
- Mapped reads to the genome using bwa mem (v0.7.17).
- Filtered mapped reads to remove low quality mappings as well as multi-mapped reads using Samtools (v1.3.1) (Danecek et al. 2021).
- Soft clipped overlapping reads not merged by FLASH using bamUtil (v1.0.15).
- Concatenated paired and unpaired reads into a single forward and reverse bam file for each sample.

- Removed PCR duplicates using Picard (v2.26.4).

▼ Software used for sequence processing and analysis

- parallel
- fastqc
- Trimmomatic
- Flash
- bwa
- Samtools
- Bamutil
- Picard
- angsd
- RStudio

We used the University of Miami's high performance computing system for our lcWGS analyses and were working in a projects directory. Within this directory we made a directory for our hardhead silverside analyses that contained a directory for the raw sequences. Other directories can be made at this time or, alternatively, can be made as part of the coding scripts. After downloading our sequences into our raw_seq directory, we made a list of the sequencing reads (i.e., the unique SRR numbers for each sample).

▼ CODE

```
# Make initial directories
mkdir -p hhsilver/raw_seq

# -p, --parents
        #no error if existing, make parent directories as needed

# After loading sequences into the raw_seq directory,
# we had paired-end reads named something similar to SRR4781607_1.fastq.gz
# and SRR4781607_2.fastq.gz for each sample

# move to the folder with the raw sequences
cd /projects/hhsilver/raw_seq

# pull out the uniqe SRR numbers using ls, grep, and cut, and write to a file
ls | grep .*_1 | cut -f 1 -d '_' > /projects/hhsilver/ids.txt
```

Trim adapters and low quality sequences

Pre and post raw sequence trimming (we're using Trimmomatic), fastqc was used to visually assess sequence quality.

▼ CODE

```
# Run fastqc on raw sequences

# Define Variables
IDS=/projects/hhsilver/ids.txt #sample SRR ids
R1=/projects/hhsilver/raw_seq/{}_1.fastq.gz
R2=/projects/hhsilver/raw_seq/{}_2.fastq.gz
FASTQC=/projects/hhsilver/fast_qc/ #fastqc output folder

# run fastqc using 15 threads
cat $IDS | parallel fastqc -t 15 -o $FASTQC $R1 $R2

##############################
# Remove adapter sequence with Trimmomatic (the NexteraPE.fa is a list of the adapter/index sequences we use
d for our libraries)
# Note that the adapter file (NexteraPE-PE.fa) has to be in the directory where you are running the script-
 i.e., where the sequences you are trimming are
# Adaptors are included with Timmomatic and can be found here: Trimmomatic-0.39/adapters

# change to directory with adapter list and sequences
cd /projects/hhsilver/raw_seq/

# make directory for the trimmed sequences
mkdir /projects/hhsilver/trimmed_seq

# Define Variables
IDS=/projects/hhsilver/ids.txt #sample SRR ids
R1=/projects/hhsilver/raw_seq/{}_1.fastq.gz
R2=/projects/hhsilver/raw_seq/{}_2.fastq.gz
R1_PAIRED=/projects/hhsilver/trimmed_seq/{}_R1.paired.fq.gz
R1_UNPAIRED=/projects/hhsilver/trimmed_seq/{}_R1.unpaired.fq.gz
R2_PAIRED=/projects/hhsilver/trimmed_seq/{}_R2.paired.fq.gz
R2_UNPAIRED=/projects/hhsilver/trimmed_seq/{}_R2.unpaired.fq.gz

# Code
cat $IDS | parallel "java -jar ~/software/src/Trimmomatic-0.39/trimmomatic-0.39.jar \
PE -threads 15 -phred33 $R1 $R2 \
$R1_PAIRED $R1_UNPAIRED $R2_PAIRED $R2_UNPAIRED \
ILLUMINACLIP:NexteraPE-PE.fa:2:30:10:4:true LEADING:10 SLIDINGWINDOW:4:15 MINLEN:50"

# this code uses 15 threads, and does the following as described:
# ILLUMINACLIP: Removes Illumina adapters provided in the NexteraPE-PE.fa file.
    # Looks for seed matches allowing maximally 2 mismatches.
    # For paired end reads, these seeds will be extended and clipped if a score of 30 is reached,
    # or in the case of single ended reads a score of 10.
    # The minimum adapter length in palindrome mode is 4.
    # True is to keep both reads in the case of a read-through.
# LEADING: Removes leading low quality or N bases (below quality 10)
# SLIDINGWINDOW: Scans the read with a 4-base wide sliding window, cutting when the average quality per base
drops below 15
# MINLEN: Drops reads which are less than 50 bases long after these steps

##############################
# Run fastqc on quality trimmed sequences

# run fastqc
cat $IDS | parallel fastqc -t 15 -o $FASTQC $R1_PAIRED $R1_UNPAIRED $R2_PAIRED $R2_UNPAIRED
```

Merge overlapping reads using FLASH

Note that flash will not work with parallel because it needs the mate-pair files to be paired together in the same order. Flash might be more appropriate to improve genome assemblies and may not be as useful here, especially since we also soft-clipped any overlapping reads after aligning the reads to the genome.

After merging overlapping paired reads into single (rather than paired) sequences, all single sequences (flash merged as well as unpaired sequences resulting from Trimmomatic) were concatenated into a single file in preparation for mapping to the genome with BWA.

▼ CODE

```
# make directory for FLASH merged output
mkdir /projects/hhsilver/flash

# Define Variables
IDS=/projects/hhsilver/ids.txt #sample SRR ids
R1_PAIRED=/projects/hhsilver/trimmed_seq/$id\_R1.paired.fq.gz
R2_PAIRED=/projects/hhsilver/trimmed_seq/$id\_R2.paired.fq.gz
FLASHOUT=/projects/hhsilver/flash

# loop through samples and run Flash
# note that the $(cat $IDS) is a command substitution and can be written as
# $(...) or `...` where $(...) is the more modern way. The purpose of command
# substitution is to evaluate the command within the parentheses or back ticks
# and provide its result as an argument to the actual command.
# Thus, everything in the parentheses is evaluated (executed)
# by the shell before the main command, and the output of that execution is
# used by that main command, just as if you'd typed that output at that place
# in the command line.

for id in $(cat $IDS)
do flash -z -o $id -d $FLASHOUT $R1_PAIRED $R2_PAIRED
done
# -o: prefix of output files; -d: path to directory for the output files.
# -z, --compress Compress the output files directly with zlib

# the flash output will include (for each sample):
            # sample.extendedFrags.fastq.gz
            # sample.notCombined_1.fastq.gz
            # sample.notCombined_2.fastq.gz

##############################
# Next, concatenate single reads into one file for each sample

# make a directory for singletons
mkdir /projects/hhsilver/singlet/{}_singlet.fastq.gz

# Define Variables
R1_UNPAIRED=/projects/hhsilver/trimmed_seq/$id\_R1.unpaired.fq.gz
R2_UNPAIRED=/projects/hhsilver/trimmed_seq/$id\_R2.unpaired.fq.gz
FLASHSINGLETS=/projects/hhsilver/flash/$id\.extendedFrags.fastq.gz
OUTFILE=/projects/hhsilver/singlet/$id\_singlet.fastq.gz

# concatenate singletons (flash output, R1_UNPAIRED, and R2_UNPAIRED) for each sample
for id in $(cat $IDS)
do cat $FLASHSINGLETS $R1_UNPAIRED $R2_UNPAIRED > $OUTFILE
done
```

Map reads to the genome and filter

We mapped our reads using BWA mem with default conditions. We first indexed the reference genome sequence and then mapped the reads.

The headers of the fastq files have information on the sequencing run that we put into the Read Group information (where a read group is the set of reads that are generated from a single run of a sequencing

instrument). Downstream programs might require this information. Furthermore, you might want to examine your data for technical artifacts (*e.g.*, to see if samples cluster based on the instrument, flow cell, or sequencing lane the sample was run on). Although our samples were run on a single sequencing lane making this information uninformative for exploring these technical artifacts, we assigned read groups to preclude potential problems with later software. Thus, we used the fastq headers to assign read groups when we mapped the reads. This is done with BWA's -R option.

We then converted the resulting sam files into bam files to save space.

▼ CODE

```
# Make a directory for the bwa output
mkdir /projects/hhsilver/singlet/bwa_out

# Define Variables
IDS=/projects/hhsilver/ids.txt #sample SRR ids
REF=/projects/refs/HH_gen.fasta
PREFIX=/projects/refs/hh_gen
R1=/projects/hhsilver/flash/{}.notCombined_1.fastq
R2=/projects/hhsilver/flash/{}.notCombined_2.fastq
SINGLETS=/projects/hhsilver/singlet/{}_singlet.fastq.gz
BWA_PAIR_OUT=/projects/hhsilver/bwa_out/{}.pair.sam
BWA_SINGLE_OUT=/projects/hhsilver/bwa_out/{}.single.sam

# Define Variables for the Read Group
HEADER=$(zcat {} | head -n 1)
ID=$(echo $HEADER | head -n 1 | cut -f 1-4 -d":" | sed 's/@//' | sed 's/:/_/g') # instrument, run #, flow ce
ll, and lane
SM=$(echo $HEADER | head -n 1 | grep -Eo "[ATGCN]+\+[ATGCN]+$") # sample index

# Define Variables for samtools
SAM_SINGLE=/projects/hhsilver/bwa_out/{}.single.sam
SAM_PAIR=/projects/hhsilver/bwa_out/{}.pair.sam
BAM_SINGLE=/projects/hhsilver/bwa_out/{}.single.bam
BAM_PAIR=/projects/hhsilver/bwa_out/{}.pair.bam

# Index the reference genome
# Build index in the reference folder
bwa -p $PREFIX index $REF

##############################
# Map the paired reads, adding a Read Group
# don't think you need the echo here, based on bwa manual
# -R STR    Complete read group header line. ’\t’ can be used in STR and will be converted to a TAB in the o
utput SAM. The read group ID will be attached to every read in the output. An example is ’@RG\tID:foo\tSM:ba
r’. [null]
cat $IDs | parallel bwa mem -t 15 -M\
-R "@RG'\'tID:$ID'\'tSM:$SM'\'tLB:$ID\_$SM'\'tPL:ILLUMINA" \
$REF $R1 $R2 > $BWA_PAIR_OUT
# -M     Mark shorter split hits as secondary (for Picard compatibility)

# Map the singlets, adding a Read Group
cat $IDs | parallel bwa mem -t 15 \
-R "@RG'\'tID:$ID'\'tSM:$SM'\'tLB:$ID\_$SM'\'tPL:ILLUMINA" -M\
$REF $SINGLETS > $BWA_SINGLE_OUT

##############################
# Convert sam files into bam files
cat $IDs | parallel samtools view -bS $SAM_PAIR > $BAM_PAIR
cat $IDs | parallel samtools view -bS $SAM_SINGLE > $BAM_SINGLE
```

Next, we used samtools to filter the mapped reads for mapping quality and non-unique mapping. We used grep for this, which is slow. An alternative such as sambaba might be a better approach.

▼ CODE

```
# make directory for filtered bam files
mkdir /projects/hhsilver/bam_filter/

# Define Variables
IDS=/projects/hhsilver/ids.txt #sample SRR ids
BAM_SINGLE=/projects/hhsilver/bwa_out/{}.single.bam
BAM_PAIR=/projects/hhsilver/bwa_out/{}.pair.bam
BAM_FILTER_SINGLE=/projects/hhsilver/bam_filter/{}.single.filter.bam
BAM_FILTER_PAIR=/projects/hhsilver/bam_filter/{}.pair.filter.bam

# filtered mapped reads for quality (-q20) and removed reads with non-unique or split mappings
# AX:Z: (alternative hits) marks multimapped reads,
# SA:Z: marks split read alignments- parts of reads aligning to multiple places

cat $IDs | parallel "samtools view -h -q20 $BAM_PAIR | grep -v -e 'XA:Z:' -e 'SA:Z:' | \
samtools view -buS - | samtools sort - -o $BAM_FILTER_PAIR"

cat $IDs | parallel "samtools view -h -q20 $BAM_SINGLE | grep -v -e 'XA:Z:' -e 'SA:Z:' | \
samtools view -buS - | samtools sort - -o $BAM_FILTER_SINGLE"

# with samtools, if you put - in place of in.bam (the input file) then samtools
# will take input for in.bam from stdin
```

After filtering our mapped reads with samtools, we used bamUtil to flag overlapping paired reads (these represent sequences from the same DNA molecule and can influence Genotype Likelihoods if not removed) and then removed these marked reads using samtools. Next, we merged the bam files (clipped paired reads and singlets [unpaired and flash merged]) into a single bam file for each sample.

▼ CODE

```
# make directory for clipped bam files and merged bam files
mkdir /projects/hhsilver/bam_clip/ /projects/hhsilver/bam_merge/

# set variables
IDS=/projects/hhsilver/ids.txt #sample SRR ids
BAM_FILTER_SINGLE=/projects/hhsilver/bam_filter/{}.single.filter.bam
BAM_FILTER_PAIR=/projects/hhsilver/bam_filter/{}.pair.filter.bam
CLIPPED_BAMS=/projects/hhsilver/bam_clip/{}_paired_clip_filt.bam
CLIPPED_MAPPED=/projects/hhsilver/bam_clip/{}_paired_clip_mapONLY_filt.bam
BAM_MERGE=/projects/hhsilver/bam_merge/{}_merge.bam


# use clipOverlap to soft-clip overlapping read ends
# clipOverlap clips the records whose clipped region has the lowest average quality.
cat $IDS | parallel bam clipOverlap --in $BAM_FILTER_PAIR --out $CLIPPED_BAMS \
--unmapped --storeOrig OC --stats
# --unmapped     : Mark records that would be completely clipped as unmapped
# --storeOrig    : Store the original cigar in the specified tag." (needs to be 2 characters s.a. OC [Origin
al CIGAR])
# --stats        : Print some statistics on the overlaps


# remove reads that became unmapped in the clipping
cat $IDS | parallel "samtools view -hb -F 4 $CLIPPED_BAMS > $CLIPPED_MAPPED"
```

```
# -F 4   do not output alignments with any bits set in FLAG present in the FLAG field
# where 4 --> segment unmapped

###########################
# concatenate the bam files (clipped paired reads and filtered singlets) for each sample
for id in $(cat $IDS)
do cat $CLIPPED_MAPPED $BAM_FILTER_SINGLE > $BAM_MERGE
done
```

Finally, we used Picard to remove duplicates. If these are not removed, sequence depth will be inflated.

▼ CODE

```
# make directory for de-duplicated files, which are the final bam files
mkdir /projects/hhsilver/final_bam/

# set variables
IDS=ids.txt
BAM_MERGE=/projects/hhsilver/bam_merge/{}_merge.bam
FINAL_BAM=/projects/hhsilver/final_bam/{}_final.bam
METRICS=/projects/hhsilver/final_bam/{}_dupstat.txt


# mark and remove duplicates with picard -- can inflate depth
cat $IDS | parallel "picard MarkDuplicates I=$BAM_MERGE O=$FINAL_BAM M=$METRICS \
VALIDATION_STRINGENCY=SILENT REMOVE_DUPLICATES=true MAX_FILE_HANDLES=1000"
```

lcWGS data analyses

After raw sequence processing, we used ANGSD [v0.935, (Korneliussen, Albrechtsen et al. 2014)] for genotype likelihood calling. Genotype likelihoods were retained for 266,731 out of 491,873,062 sites in 75 individuals after filtering.

▼ filtering parameters

- minimum depth=15
- maximum depth=750
- base quality >20
- mapping quality >20
- minor allele frequency >0.1
- SNP p-value <1e-6
- SNP coverage in at least 35 individuals
- Additional arguments recommended for bams mapped with bwa also were used:
    - remove_bads 1
    - only_proper_pairs 1
    - C 50

▼ CODE

```
# Angsd requires a filelist containing the full path for each bam file with one
# filename per row. For us, this list contained entries something like
# /projects/hhsilver/final_bam/SRR4781607_final.bam

# this list can be generated by moving to the directory with your final bam
# files and using code similar to the following:
ls | grep -e .*_final.bam$ | sed -r 's/^/\/projects\/hhsilver\/final_bam\//' \
> /projects/hhsilver/final_bam/bam_list.txt

# make directory for angsd output
mkdir /projects/hhsilver/angsd/

# set variables
BAM_LIST=/projects/hhsilver/final_bam/bam_list.txt
ANGSD=~/software/local/angsd/angsd # path the angsd software
OUTFILE=/projects/hhsilver/angsd
REF=/projects/refs/HH_gen.fasta

# run ANGSD to get outputs: genotype likelihood file, MAF file, counts file,
# genotype file, depth file, counts file, beagle file, vcf file
# for information on specific options, type ./angsd METHOD, e.g., ./angsd -GL

# set arguments
MINDP=15       # Minimum depth filter
MAXDP=750      # Maximum depth filter
MININD=35      # Minimum individual filter
MINQ=20      # Minimum quality filter
MINMAF=0.1   # Minimum minor allele frequency filter
MINMAPQ=20   # Minimum mapping quality (alignment score) filter, default value is 20
BAQ=1        # Base alignment quality; to avoid INDEL alignment artifacts in low-coverage multi-sample SNP c
alling; -dobaq 1 is standard baq and -dobaq 2 is extended baq; 0: No BAQ calcualtion
SNP='1e-6'     # only work with sites with a p-value less than [float]; requires -doMaf
EXTRA_ARG='-remove_bads 1 -only_proper_pairs 1 -C 50' # Extra arguments when running ANGSD
# -remove_bads  Same as the samtools flags -x which removes read with a flag above 255
# -only_proper_pairs  Include only proper pairs (pairs of read with both mates mapped correctly)
# -C 50  Adjust mapQ for excessive mismatches

# run angsd to calculate genotype likelihoods
cd $BAM_DIR
$ANGSD -bam $BAM_LIST -ref $REF -out $OUTFILE/hh -GL 1 \
-doGlf 2 -doBcf 1 -doMaf 1 -doMajorMinor 1 -doPost 1 -doCounts 1 -doDepth 1 \
-baq $BAQ -maxDepth $MAXDP -dumpCounts 1 -doIBS 1 -makematrix 1 -doCov 1 \
-setMinDepth $MINDP -setMaxDepth $MAXDP -minInd $MININD -minQ $MINQ \
-minMapQ $MINMAPQ -SNP_pval $SNP -minMaf $MINMAF $EXTRA_ARG -nThreads 15

# -GL 1 use SAMtools liklihood, as opposed to GATK (2)
# -doGlf 2 Output the log genotype likelihoods to a file; beagle genotype likelihood format (2)
# -doBcf 1 still beta; wrapper around -gl -domajorminor and -dopost -dogeno
# -doMaf 1 1: Known major, and Known minor. Here both the major and minor allele is assumed to be known (inf
erred or given by user). The allele frequency is the obtained using based on the genotype likelihoods.
# -doMajorMinor 1 From input for either sequencing data like bam files or from genotype likelihood data like
glfv3 the major and minor allele can be inferred directly from likelihoods. We use a maximum likelihood appr
oach to choose the major and minor alleles.
# -doPost 1 calculate posterior maf probabilities; 1: Using frequency as prior
# -doCounts 1 Calculate various counts statistics
# -doDepth 1 This method will find depth distribution for every sample and for all samples jointly.
# -dumpCounts 1 1: Print overall depth in the .pos file. This depth is the sum of reads covering a sites for
all individuals. The first column is the chromosome, the second it the position the third is the total dept
h. 2: prints the depth of each individual.
# -doIBS 1 Identity by state matrix (average proportion of alleles shared by 2 individuals across the autoso
mal genome;
# -makematrix 1 print out the ibs matrix
# -doCov 1 print out the cov matrix (from doIBS)
```

We used PCAngsd (Meisner and Albrechtsen 2018) to analyze the resulting beagle file. PCAngsd estimates the covariance matrix and individual allele frequencies for low-depth sequencing data and uses principal component analysis (PCA) to perform multiple population genetic analyses using genotype likelihoods. Once we got the covariance matrix, we graphed it in RStudio.

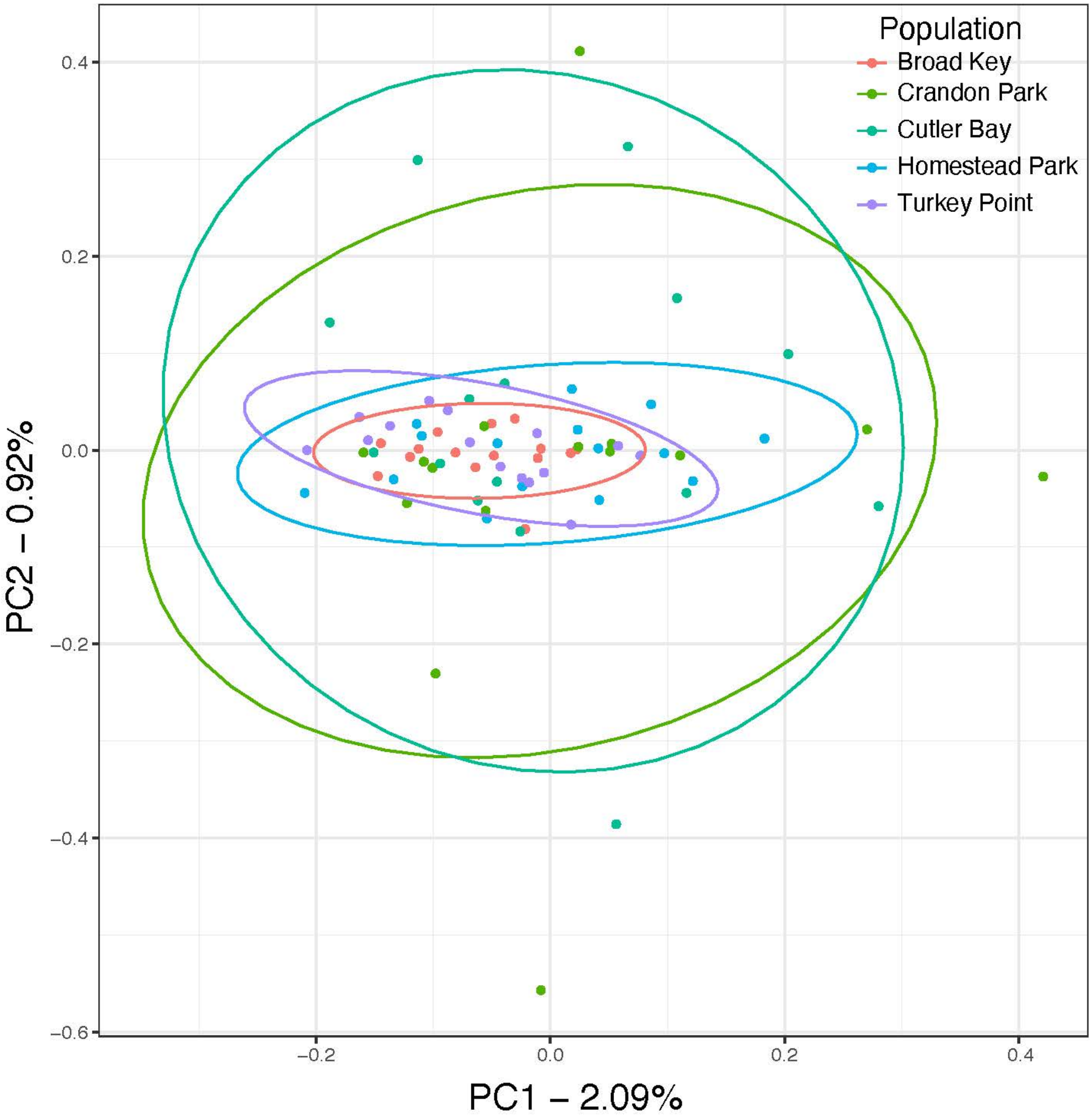

▼ **CODE**

```
# define variables
BGL=/projects/hhsilver/angsd/hh.beagle.gz
```

```
OUTFILE=/projects/hhsilver/angsd
PCA=~/software/pcangsd/pcangsd.py # path to pcangsd software

# run PCAngsd
python $PCA -beagle $BGL -out $OUTFILE/hh_pca -threads 15
```

The R code and metadata for the PCA figure is below.

▼ R CODE

```
# load libraries
library(ggplots2)
library(dplyr)
library(tidyr)

# Output from PCAngsd .cov file
covs <- as.matrix(read.table("~/Desktop/wg_analysis/astipes/hh_pca.cov")) # Reads in estimated covariance ma
trix
covs <- as.data.frame(covs)

# get eigenvalues and vectors
covs_eigen <- eigen(covs)
cov_eigen_vals <- covs_eigen$values
cov_eigen_vecs <- as.data.frame(covs_eigen$vectors)

# Read in metadata and add column names for merging
metadata <- read.csv("HHsilverside_WGS_samplesheet.csv")

# list of samples in order of bam file list
samps <- read.csv("num_samples.csv", header=FALSE)
colnames(samps) <- c("num","sample_id")

# remove Reef fish - samples not used
samps_noR <- subset(samps, samps$sample_id!=c("R-124","R-125","R-127","R-128","R-130"))

# combine sampleIds with covariance dataframe
eigen_full <- cbind(cov_eigen_vecs, samps_noR)

# Merge with other with metadata based on shared sample_id column
eigen_full <- full_join(metadata, eigen_full, by="sample_id")

# use ggplot for plotting - Figure 4
ggplot(data=eigen_full) +
  geom_point(aes(x=V1, y=V2, col=population)) +
  labs(x="PC1 - 2.09%", y="PC2 - 0.92", title="PCAngsd - ~266k nuclear GL") +
  stat_ellipse(aes(x=V1, y=V2, col=population), size=0.5) +
  theme_bw()

# plot percent variance explained by top 10 PCs - Figure S1
sum(cov_eigen_vals[1:10])
barplot(cov_eigen_vals[1:10])
```

sample_id	population	species
BK-01	Broad-Key	hardhead
BK-02	Broad-Key	hardhead
BK-03	Broad-Key	hardhead
BK-04	Broad-Key	hardhead

BK-05	Broad-Key	hardhead
BK-06	Broad-Key	hardhead
BK-07	Broad-Key	hardhead
BK-08	Broad-Key	hardhead
BK-09	Broad-Key	hardhead
BK-10	Broad-Key	hardhead
BK-25	Broad-Key	hardhead
BK-26	Broad-Key	hardhead
BK-27	Broad-Key	hardhead
BK-30	Broad-Key	hardhead
BK-31	Broad-Key	hardhead
CP-20	Crandon-Park	hardhead
CP-02	Crandon-Park	hardhead
CP-03	Crandon-Park	hardhead
CP-04	Crandon-Park	hardhead
CP-07	Crandon-Park	hardhead
CP-08	Crandon-Park	hardhead
CP-10	Crandon-Park	hardhead
CP-11	Crandon-Park	hardhead
CP-13	Crandon-Park	hardhead
CP-14	Crandon-Park	hardhead
CP-15	Crandon-Park	hardhead
CP-16	Crandon-Park	hardhead
CP-17	Crandon-Park	hardhead
CP-18	Crandon-Park	hardhead
CP-19	Crandon-Park	hardhead
M-01	M61	hardhead
M-02	M61	hardhead
M-03	M61	hardhead
M-04	M61	hardhead
M-05	M61	hardhead
M-06	M61	hardhead
M-07	M61	hardhead
M-08	M61	hardhead
M-09	M61	hardhead
M-10	M61	hardhead
M-11	M61	hardhead

M-12	M61	hardhead
M-13	M61	hardhead
M-14	M61	hardhead
M-15	M61	hardhead
M-22	M87	hardhead
M-23	M87	hardhead
M-24	M87	hardhead
M-25	M87	hardhead
M-26	M87	hardhead
M-27	M87	hardhead
M-28	M87	hardhead
M-29	M87	hardhead
M-30	M87	hardhead
M-31	M87	hardhead
M-32	M87	hardhead
M-33	M87	hardhead
M-34	M87	hardhead
M-35	M87	hardhead
M-36	M87	hardhead
M-61	M26	hardhead
M-62	M26	hardhead
M-63	M26	hardhead
M-64	M26	hardhead
M-65	M26	hardhead
M-66	M26	hardhead
M-67	M26	hardhead
M-68	M26	hardhead
M-69	M26	hardhead
M-70	M26	hardhead
M-71	M26	hardhead
M-72	M26	hardhead
M-73	M26	hardhead
M-74	M26	hardhead
M-75	M26	hardhead
R-124	Reef	reef
R-125	Reef	reef
R-127	Reef	reef

R-128	Reef	reef
R-130	Reef	reef

**rownumber**	**sample_id**
1	BK-01
2	BK-02
3	BK-03
4	BK-04
5	BK-05
6	BK-06
7	BK-07
8	BK-08
9	BK-09
10	BK-10
11	BK-25
12	BK-26
13	BK-27
14	BK-30
15	BK-31
16	CP-02
17	CP-03
18	CP-04
19	CP-07
20	CP-08
21	CP-10
22	CP-11
23	CP-13
24	CP-14
25	CP-15
26	CP-16
27	CP-17
28	CP-18
29	CP-19
30	CP-20
31	M-01
32	M-02
33	M-03

34	M-04
35	M-05
36	M-06
37	M-07
38	M-08
39	M-09
40	M-10
41	M-11
42	M-12
43	M-13
44	M-14
45	M-15
46	M-22
47	M-23
48	M-24
49	M-25
50	M-26
51	M-27
52	M-28
53	M-29
54	M-30
55	M-31
56	M-32
57	M-33
58	M-34
59	M-35
60	M-36
61	M-61
62	M-62
63	M-63
64	M-64
65	M-65
66	M-66
67	M-67
68	M-68
69	M-69
70	M-70

71	M-71
72	M-72
73	M-73
74	M-74
75	M-75
76	R-124
77	R-125
78	R-127
79	R-128
80	R-130
