# Supplementary material for: Sequencing Bait: Nuclear and Mitogenome Assembly of an Abundant Coastal Tropical and Subtropical Fish, Atherinomorus stipes
Source: Genome Biol Evol. 2022 Jul 22;14(8):evac111. doi: 10.1093/gbe/evac111 (PMC9348626; doi:10.1093/gbe/evac111)
Supplement: evac111_Supplementary_Data [file evac111_supplementary_data.zip › Hardhead_Supplemental_Figures_revised.docx]

# Supplemental Figures

**Figure S1: Gene duplication events among fish species.** Phylogenetic tree shows relationships between species with the number of shared ancestral gene duplication events at each node and number of unique gene duplication events for a given species at each tip. Visualized using Orthofinder.


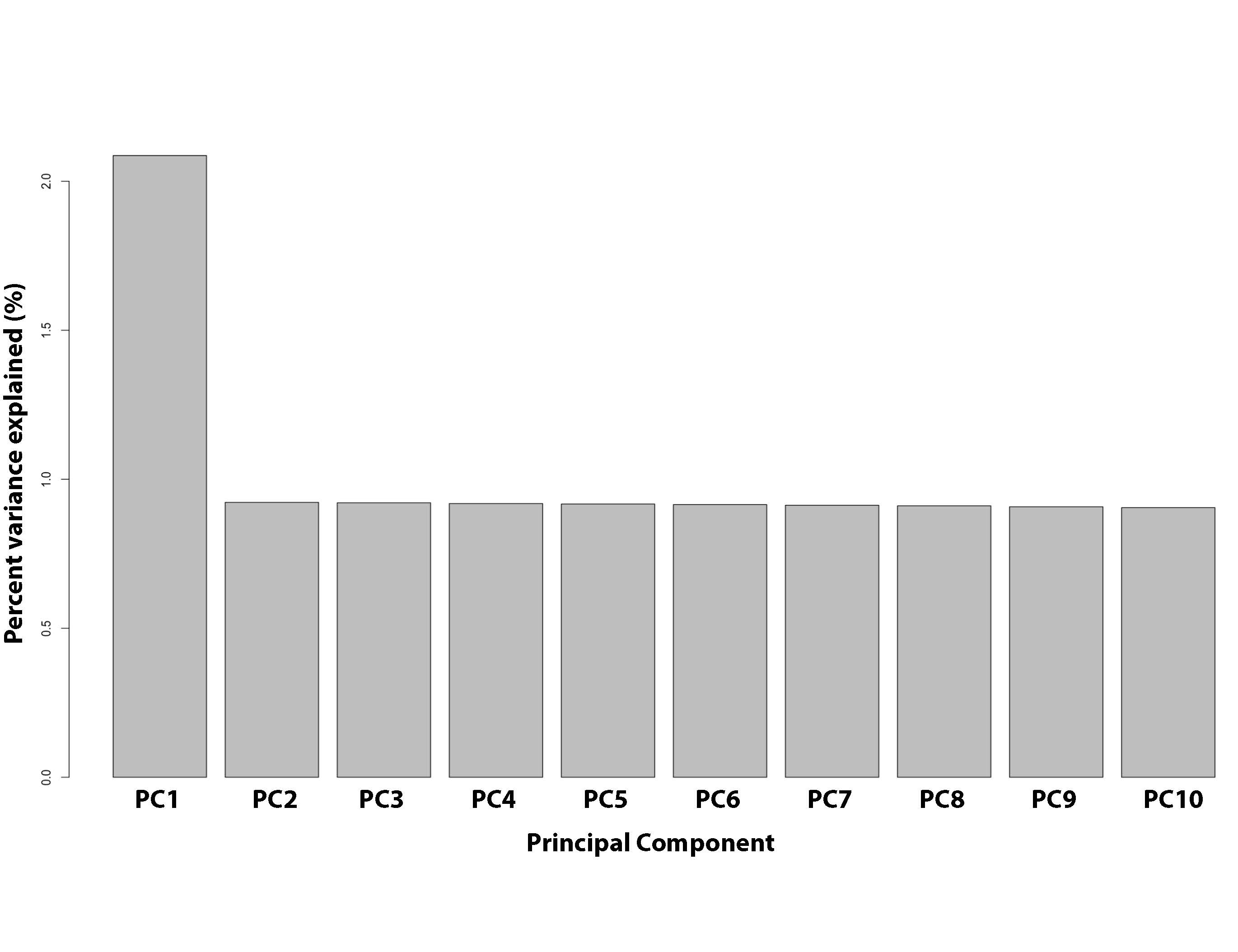


**Figure S2: Percent variance explained among principal components.** Variance explained (y-axis) by the first ten principal components (PC, x-axis). Sum of the first ten principal components is 10.32%.
